# Differences in Oral Function, Masticatory Movement, and MRI-Based Structural Features of the Temporomandibular Joint Between the Deviated and Non-Deviated Sides in Patients with Facial Asymmetry

**DOI:** 10.3390/diagnostics16091274

**Published:** 2026-04-23

**Authors:** Rintaro Kubo, Syunnosuke Toyama, Yudai Shimpo, Kiichiro Mizokami, Mari Kaneda, Minami Seki, Hiroshi Tomonari

**Affiliations:** Department of Orthodontics, Tsurumi University School of Dental Medicine, Yokohama 230-8501, Japan; rin.k.regulus@gmail.com (R.K.); shinpo-y@tsurumi-u.ac.jp (Y.S.); earth3120@gmail.com (K.M.); kaneda-m@tsurumi-u.ac.jp (M.K.); ssaw0209@icloud.com (M.S.); tomonari-h@tsurumi-u.ac.jp (H.T.)

**Keywords:** facial asymmetry, dentofacial deformity, masticatory movement, oral function, temporomandibular joint, magnetic resonance imaging, chewing side, functional assessment

## Abstract

**Background:** Facial asymmetry in patients with dentofacial deformity is often associated with occlusal asymmetry, functional differences in mastication, and temporomandibular joint (TMJ) conditions. However, side-to-side differences in the stability of masticatory movement and their relationship with oral function have not been fully clarified. Therefore, this study aimed to investigate differences in masticatory movement, oral function, and the prevalence of MRI-based structural features of the temporomandibular joint between the deviated and non-deviated sides in patients with facial asymmetry. **Methods:** Twenty-one patients with dentofacial deformity and facial asymmetry were included in this study. Oral function was evaluated by measuring occlusal contact area, occlusal force, and masticatory performance. Masticatory movement was recorded using a mandibular movement recording system, and parameters related to the masticatory path and chewing speed were calculated. The stability of masticatory movement was evaluated using the variance of these parameters across chewing cycles. Temporomandibular joint structural features were assessed using CT and MRI. Comparisons between the deviated and non-deviated sides were performed using paired statistical tests. **Results:** Occlusal contact area and occlusal force were significantly greater on the deviated side than on the non-deviated side (*p* = 0.001 and *p* = 0.006, respectively), whereas no significant difference was observed in masticatory performance (*p* = 0.211). The deviated side showed a smaller closing angle (*p* = 0.005) and maximum lateral amplitude (*p* = 0.019), indicating a more vertical chewing pattern. The stability of masticatory movement, evaluated using the variance of masticatory path and velocity parameters, was significantly greater on the deviated side (e.g., variance of cycle axis angle, *p* = 0.002; variance of maximum closing velocity, *p* = 0.006). In addition, the prevalence of imaging-based structural features of the temporomandibular joint was significantly higher on the deviated side (*p* = 0.016). **Conclusions:** Patients with dentofacial deformity and facial asymmetry were associated with functional asymmetry between the deviated and non-deviated sides. The deviated side showed greater occlusal contact area, occlusal force, and stability of masticatory movement. These findings suggest that the deviated side may be functionally favorable for mastication and may be related to a tendency toward preferential chewing behavior. However, because the habitual chewing side was not directly evaluated in the present study, this interpretation should be considered cautiously and viewed as hypothesis-generating.

## 1. Introduction

Dentofacial deformity with facial asymmetry is one of the important clinical problems in the oral and maxillofacial region, not only because of esthetic concerns but also because of functional disturbances such as masticatory dysfunction and speech impairment caused by malocclusion [1,2,3]. In patients with facial asymmetry, occlusal asymmetry associated with skeletal and dental discrepancies between the left and right sides has been reported, resulting in side-dependent differences in occlusal contact conditions and the functional environment during mastication [4]. Therefore, in such patients, it is important to assess not only morphological characteristics but also oral function, masticatory movement, and temporomandibular joint status. Orthodontic approaches, including the use of intermaxillary elastics, have been widely applied in the management of skeletal discrepancies and malocclusions, contributing to both functional and morphological improvements [5].

Previous studies have reported differences between the deviated and non-deviated sides in occlusal contact area, occlusal force, and the prevalence of temporomandibular joint structural features assessed using imaging modalities in patients with facial asymmetry [6,7,8,9,10]. In addition, masticatory movement is known to be influenced not only by morphological characteristics such as occlusal support and skeletal deviation but also by sensory input from periodontal mechanoreceptors and other peripheral receptors [11,12,13].

It has been reported that favorable occlusal relationships and stable occlusal support are associated with improved stability of masticatory movement and more efficient masticatory muscle activity, suggesting that side-to-side differences in occlusal contact area and occlusal force may be reflected as differences in masticatory rhythm and masticatory path [14,15,16,17]. Therefore, it is conceivable that in patients with facial asymmetry, side-dependent differences in oral function affect masticatory movement.

Although previous studies have evaluated oral function and masticatory movement in patients with anteroposterior skeletal discrepancies and in healthy individuals [18,19], reports on functional laterality in patients with facial asymmetry have mainly focused on occlusal force, occlusal contact area, or temporomandibular joint structural features, and studies investigating the stability of masticatory movement between the deviated and non-deviated sides remain limited [2]. In particular, few studies have evaluated indicators of masticatory movement, such as the stability of masticatory rhythm and masticatory path, together with oral functions including occlusal contact area, occlusal force, and masticatory performance, as well as the prevalence of temporomandibular joint structural features assessed using imaging modalities, on both the deviated and non-deviated sides in patients with facial asymmetry.

Previous studies have reported side-related differences in masticatory function, including occlusal force and muscle activity. Although quantitative data remain limited, a computational modeling study has suggested that muscle forces on the non-working side may be approximately 14% lower than those on the working side [20]. However, quantitative evaluations of the stability of masticatory movement, particularly using variability- or variance-based indicators, remain scarce. Therefore, the novelty of this study lies in the comprehensive and side-specific assessment of both oral function and the stability of masticatory movement on each side in patients with facial asymmetry.

Accordingly, the nature of functional asymmetry in patients with facial asymmetry and its relationship with occlusion and temporomandibular joint structural features assessed by imaging modalities have not yet been fully clarified. Therefore, the aim of this study was to investigate differences in masticatory movement, oral function, and MRI-based structural features of the temporomandibular joint (TMJ) between the deviated and non-deviated sides in patients with dentofacial deformity with facial asymmetry.

Specifically, we compared masticatory rhythm stability, the stability and pattern of the masticatory path, occlusal contact area, occlusal force, masticatory performance, and the prevalence of MRI-based structural features of the temporomandibular joint to clarify the characteristics of functional asymmetry in patients with facial asymmetry.

In this study, temporomandibular joint characteristics were evaluated as structural features identified on imaging and were not intended to represent clinical diagnoses of temporomandibular disorders.

## 2. Materials and Methods

### 2.1. Selection Criteria and Ethics

**Figure 1:**
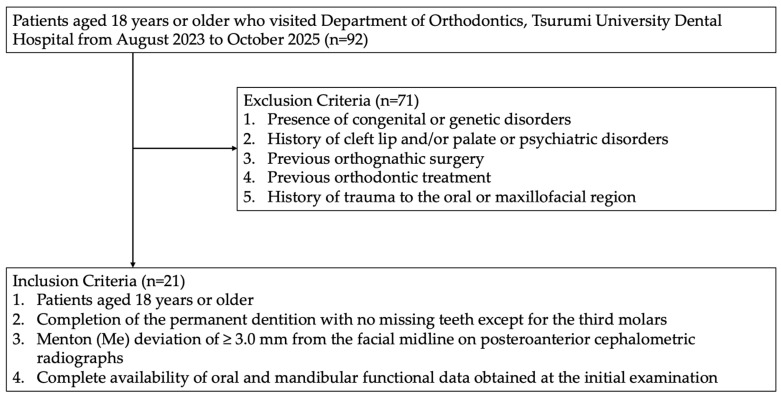
Flowchart of patient selection. Among 92 patients diagnosed with dentofacial deformity between August 2023 and October 2025, patients were screened according to the inclusion and exclusion criteria. Finally, 21 patients with facial asymmetry were included in the analysis.

The study subjects were selected from 92 patients diagnosed with dentofacial deformity at the Department of Orthodontics, Tsurumi University Dental Hospital between August 2023 and October 2025. Seventy-one patients were excluded according to the exclusion criteria, and 21 patients who met the inclusion criteria were finally included in the analysis (Figure 1).

The inclusion criteria were as follows: (1) age 18 years or older; (2) complete permanent dentition with no missing teeth except for the third molars; (3) a distance of 3.0 mm or greater between the facial midline and Menton (Me) on posteroanterior cephalograms; and (4) availability of complete oral functional and jaw functional data obtained at the initial examination.

The deviated side was defined as the side toward which the Menton (Me) was displaced relative to the facial midline on posteroanterior cephalograms, corresponding to the direction of mandibular deviation, and the contralateral side was defined as the non-deviated side [21,22]. Facial asymmetry in the present study was operationally defined based on mandibular deviation represented by menton displacement and did not differentiate between maxillary asymmetry or combined dentofacial asymmetry.

However, this classification was based solely on skeletal deviation (menton position) and did not incorporate additional occlusal or dental parameters such as dental midline discrepancies, occlusal cant, or unilateral crossbite.

The exclusion criteria were as follows: (1) congenital or hereditary disorders; (2) cleft lip, cleft palate, or psychiatric disorders; (3) a history of orthognathic treatment; (4) a history of orthodontic treatment; and (5) a history of trauma to the oral or facial region [22,23].

Based on these criteria, 21 patients with facial asymmetry (9 males and 12 females) were finally included in the analysis. The mean age of the participants was 23.5 ± 5.0 years (range, 18–35 years).

This study was approved by the Ethics Committee of Tsurumi University School of Dental Medicine (approval number: 123014) and was conducted in accordance with the Declaration of Helsinki. This retrospective observational study used clinical records and imaging data obtained during routine clinical examinations. Written informed consent for the use of clinical data for research purposes was obtained from all participants, and all data were anonymized prior to analysis.

### 2.2. Measurement of Oral Function (Occlusal Contact Area, Occlusal Force, and Masticatory Performance)

Oral function was assessed using a pressure-sensitive film occlusal analysis system (Dental Prescale II; GC Corp., Tokyo, Japan). The participants were instructed to perform maximal clenching in the intercuspal position for 3 s, and the occlusal contact condition of the entire dentition was recorded. Occlusal force and occlusal contact area were analyzed, and the obtained data were classified into the deviated and non-deviated sides based on the direction of menton deviation in each patient.

Masticatory performance was evaluated using color-changeable chewing gum (Chew Check Gum; Oral Care Inc., Tokyo, Japan). After rinsing for 15 s, the participants were instructed to chew the gum 60 times, after which the gum was flattened into a circular shape. The degree of color change was used as an indicator of masticatory performance and was measured using a colorimeter (CR-20; Konica Minolta, Tokyo, Japan) [24].

Measurements were performed three times in the following order: free chewing, right-side chewing, and left-side chewing, with a 3 min rest interval between each trial.

For analysis, right-side and left-side chewing were reclassified as chewing on the deviated side and chewing on the non-deviated side based on the direction of menton deviation in each patient. Specifically, the side corresponding to the direction of menton displacement on posteroanterior cephalograms was defined as the deviated side, and the contralateral side was defined as the non-deviated side.

The difference in chewing protocols reflects the distinct objectives of the assessments. For masticatory performance, a fixed number of chewing cycles was used to standardize the degree of food comminution. In contrast, masticatory movement was recorded over a fixed duration to capture continuous chewing behavior and to allow analysis of multiple chewing cycles for movement variability. This approach is consistent with previous studies that have adopted cycle-based protocols for masticatory performance and time-based recordings for movement analysis.

### 2.3. Measurement of Masticatory Movement

Masticatory movement was measured using a mandibular movement recording device (WinJaw; Zebris Medical GmbH, Isny, Germany). Chewing gum (Free Zone Gum Lemon Flavor; Lotte Co., Tokyo, Japan) was used as the test food. After the gum had been sufficiently softened, the participants were instructed to chew for 30 s on each side.

The recorded mandibular movement data were analyzed using original software developed at Tsurumi University, which converted the raw data obtained from the WinJaw system into coordinate data and calculated angular and distance parameters for each chewing cycle and their mean values. The analysis procedures were based on standard kinematic principles used in mandibular movement analysis, and all parameters were defined consistently according to established methods commonly used in previous studies of mandibular movement analysis.

Unstable movements immediately after the onset of chewing were excluded, as were cycles in which the starting and ending points of the chewing cycle did not coincide. Ten chewing cycles were then extracted for analysis.

**Figure 2:**
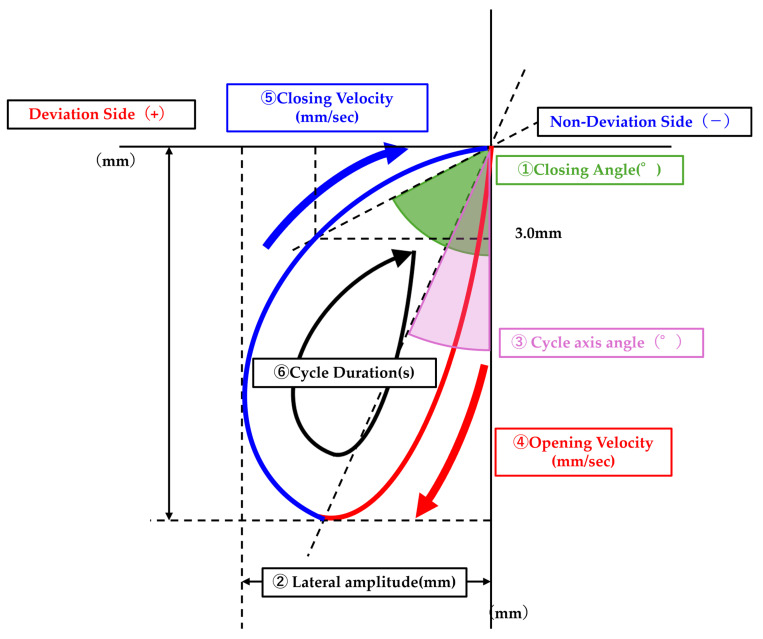
Definition of masticatory movement parameters analyzed in this study. Parameters of the masticatory path included (1) closing angle, (2) max lateral amplitude, and (3) cycle axis angle. Path stability was evaluated using the variance of closing angle, max lateral amplitude, and cycle axis angle across chewing cycles. Parameters of chewing speed included (4) max closing velocity, (5) max opening velocity, and (6) cycle duration. Velocity stability was evaluated using the variance of max closing velocity, max opening velocity, and cycle duration across chewing cycles. For all parameters, values were calculated separately for chewing on the deviated and non-deviated sides as defined based on the direction of menton deviation in each patient.

In the analysis of masticatory movement, parameters related to the masticatory path and chewing speed were evaluated (Figure 2). For the masticatory path, (1) closing angle, (2) max lateral amplitude, and (3) cycle axis angle were calculated.

The stability of the masticatory path was defined as the variability of these parameters across chewing cycles, with smaller variance indicating greater stability, consistent with previous studies that have used variability measures such as standard deviation and coefficient of variation as indicators of masticatory movement stability [25,26]: variance of closing angle, variance of max lateral amplitude, and variance of cycle axis angle.

For chewing speed, (4) max closing velocity, (5) max opening velocity, and (6) cycle duration were calculated. The stability of chewing speed was similarly defined as the variability across chewing cycles and evaluated using the variance of these parameters: variance of max closing velocity, variance of max opening velocity, and variance of cycle duration.

Each parameter was compared between chewing on the deviated side and chewing on the non-deviated side.

### 2.4. CT and MRI Examination and Evaluation

The structural features of the temporomandibular joint were evaluated based on imaging findings. Condylar deformity was assessed using CT, whereas disc position and disc dynamics were evaluated using magnetic resonance imaging (MRI).

Head and neck CT was performed using a multidetector CT scanner (Supria; Fujifilm Corp., Tokyo, Japan) at 120 kV and 60 mA. The scan range extended from the nasion to the submental region, and the reconstructed slice thickness was 0.625 mm. Images were exported in DICOM format and analyzed using image analysis software (Virtual Place Fujin; Canon Medical Systems, Tochigi, Japan).

MRI was performed using a 0.4-T open MRI system (APERTO Inspire; Fujifilm Medical Co., Tokyo, Japan) equipped with a 10.0 cm surface coil for the temporomandibular joint. The MRI protocol for the temporomandibular joint consisted of the following four sequences:

(1) Sagittal images in the intercuspal position (T2*): repetition time (TR), 400 ms; echo time (TE), 14 ms; flip angle, 45 degrees; bandwidth, 8.0 kHz; matrix size, 256 × 192; field of view (FOV), 120 mm; slice thickness, 4 mm; 9 slices per side; scan time, 5 min 9 s per side.
(2) Coronal images in the intercuspal position (T2*): TR, 400 ms; TE, 14 ms; flip angle, 45 degrees; bandwidth, 10.0 kHz; matrix size, 256 × 192; FOV, 120 mm; slice thickness, 4 mm; 9 slices per side; scan time, 5 min 23 s per side.
(3) Sagittal T2-weighted images: TR, 4400 ms; TE, 100 ms; flip angle, 90 degrees; bandwidth, 12.5 kHz; matrix size, 192 × 192; FOV, 120 mm; slice thickness, 5 mm; 6 slices per side; scan time, 3 min 36 s per side.
(4) Sagittal images in the open-mouth position (T1-weighted): TR, 1100 ms; TE, 30 ms; flip angle, 90 degrees; bandwidth, 36.5 kHz; matrix size, 256 × 192; FOV, 120 mm; slice thickness, 5 mm; 12 slices in total; scan time, 3 min 33 s.

All sequences were obtained with an FOV of 120 mm, ensuring complete coverage of the bilateral temporomandibular joint regions. During MRI acquisition, the participants were positioned supine. In the closed-mouth position, they were instructed to maintain their natural occlusion. In the open-mouth position, the mandible was supported with a bite block to maintain a stable open-mouth position during imaging.

Imaging data used in this study were obtained as part of routine clinical examinations and were analyzed to characterize structural features of the temporomandibular joint, rather than to establish clinical diagnoses of temporomandibular disorders, which require comprehensive clinical assessment in addition to imaging.

**Figure 3:**
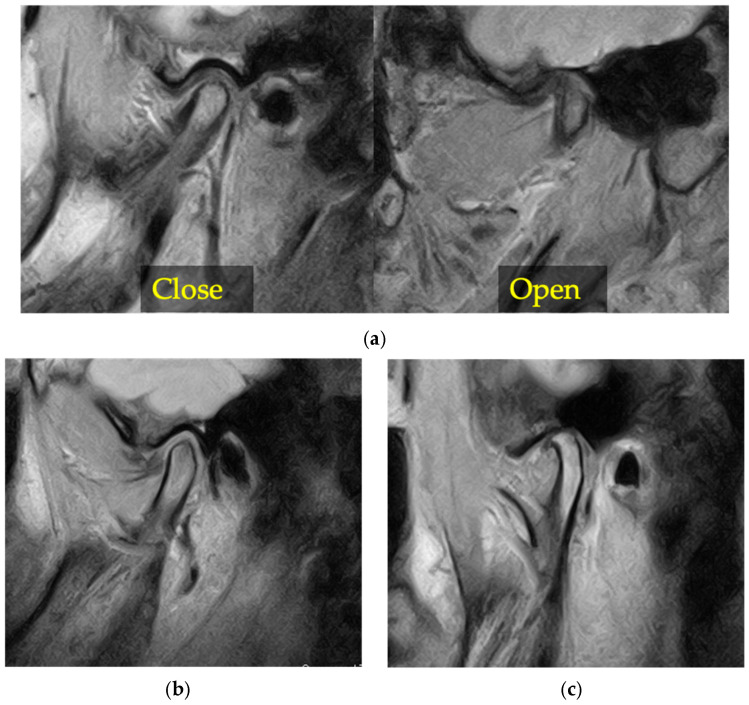
Representative MRI findings of temporomandibular joint structural features. (**a**) Disc displacement with reduction showing anterior disc displacement in the closed-mouth position and reduction of the disc in the open-mouth position. (**b**) Disc displacement without reduction showing persistent anterior disc displacement in both closed- and open-mouth positions. (**c**) Osteoarthritic changes showing condylar deformity and joint space irregularity. These images are presented for qualitative illustration of structural features and are not intended for quantitative measurement.

MRI-based structural features of the temporomandibular joint were assessed on the basis of imaging findings of disc displacement with reduction, disc displacement without reduction, and osteoarthritic changes, and their prevalence was compared between the deviated and non-deviated sides (Figure 3). These findings represent structural features identified on imaging and do not imply pathological conditions or constitute a comprehensive clinical diagnosis of temporomandibular disorders, as such diagnoses require both clinical and imaging assessments according to established diagnostic criteria.

The interpretation of CT and MRI findings was performed by experienced clinicians, including a board-certified oral and maxillofacial radiologist, according to established imaging criteria described in previous studies [27,28,29,30,31]. These imaging protocols have been used in prior clinical studies and have demonstrated acceptable reliability in identifying disc displacement and osseous changes.

### 2.5. Sample Size Calculation

The sample size in this study was determined based on previous studies on mandibular movement analysis. In a study evaluating mandibular and masticatory movement, an a priori power analysis based on preliminary data reported an effect size corresponding to a correlation coefficient of approximately r = −0.59 for the primary outcome, and the required sample size was calculated to be approximately 20 under an alpha level of 0.05 and a power of 0.80 [32].

In addition, previous studies investigating side-to-side or between-group differences in oral functions such as masticatory performance, occlusal contact area, and occlusal force were also considered when determining the sample size. Studies evaluating masticatory performance have reported effect sizes ranging from r = 0.44 to 0.87, which correspond approximately to Cohen’s d values of 1.0 to 3.0 when converted using the formula d = 2r/√(1 − r^2^) [33]. Studies examining side-to-side differences in occlusal contact area and occlusal force have also reported moderate to large effect sizes [34].

Although relatively large effect sizes were reported in these previous studies, a conservative effect size of d = 0.7 was assumed in the present study to avoid overestimation. Because the primary analysis in this study involved within-subject comparisons between the two sides, an a priori power analysis was performed using G*Power software version 3.1 (Heinrich Heine University Düsseldorf, Düsseldorf, Germany) based on a two-tailed paired *t*-test. Under an alpha level of 0.05 and a statistical power of 0.80, the required sample size was calculated to be approximately 19. Therefore, the target sample size was set at 20 participants. Since 21 participants were ultimately included in the final analysis, the sample size was considered sufficient for the planned statistical analyses. In addition, a post hoc power analysis for the primary outcomes, including variance-based indicators of masticatory movement, confirmed that the statistical power exceeded 0.80.

### 2.6. Statistical Analysis

All measurement data are presented as mean ± standard deviation. For the stability indices of masticatory movement, the variance across 10 chewing cycles was calculated for each participant. Comparisons between the deviated and non-deviated sides were treated as paired data within the same subject, and the normality of the differences was assessed using the Shapiro–Wilk test. Variables showing normal distribution were analyzed using the paired *t*-test, whereas variables not showing normal distribution were analyzed using the Wilcoxon signed-rank test. The specific statistical test applied to each variable is indicated in the corresponding tables for clarity. Differences in the presence or absence of MRI-based structural features of the temporomandibular joint between the deviated and non-deviated sides were evaluated using McNemar’s test.

To account for multiple comparisons across all statistical tests (*n* = 16), the false discovery rate (FDR) was controlled using the Benjamini–Hochberg procedure, and adjusted *p*-values (q-values) were calculated. Both unadjusted *p*-values and FDR-adjusted *p*-values (q-values) are reported.

A *p*-value < 0.05 was considered statistically significant. Statistical analyses were performed using SPSS software version 27 (IBM Corp., Armonk, NY, USA).

The dataset analyzed in this study is provided in the Supplementary Materials (File S1).

## 3. Results

### 3.1. Oral Function

Table 1 shows the comparison of oral function between the deviated and non-deviated sides. Occlusal contact area and occlusal force were significantly greater on the deviated side than on the non-deviated side (*p* = 0.001, q = 0.016; *p* = 0.006, q = 0.019, respectively), whereas no significant difference was observed in masticatory performance (*p* = 0.211, q = 0.338).

Specifically, the deviated side showed approximately 40–45% greater occlusal contact area and approximately 25–30% greater occlusal force than the non-deviated side.

The effect sizes for occlusal contact area and occlusal force were moderate to large (Cohen’s d = 0.91 and 0.67, respectively), indicating that the observed differences are not only statistically significant but also of potential clinical relevance.

### 3.2. Path and Velocity of Masticatory Movement

#### 3.2.1. Path and Path Stability of Masticatory Movement

Table 2 shows the comparison of the masticatory path and path stability between the deviated and non-deviated sides. Closing angle and max lateral amplitude were significantly smaller on the deviated side than on the non-deviated side (*p* = 0.005, q = 0.019; *p* = 0.019, q = 0.043, respectively), whereas no significant difference was observed in the cycle axis angle.

In addition, the variance of the cycle axis angle was significantly smaller on the deviated side (*p* = 0.002, q = 0.016), indicating greater stability of the masticatory path. The deviated side showed a substantially lower variability in the cycle axis angle (approximately 40% reduction).

In contrast, no significant differences were found in the variance of the closing angle or the variance of the max lateral amplitude.

The observed effect sizes for selected parameters (e.g., closing angle and variance of cycle axis angle) were moderate, suggesting meaningful side-to-side differences in masticatory movement characteristics.

#### 3.2.2. Velocity and Velocity Stability of Masticatory Movement

Table 3 shows the comparison of masticatory velocity and velocity stability between the deviated and non-deviated sides. No significant differences were observed in the mean values of max closing velocity, max opening velocity, or cycle duration between the two sides.

However, the variance of max closing velocity was significantly smaller on the deviated side (*p* = 0.006, q = 0.019), corresponding to an approximately 35–40% reduction in variability compared with the non-deviated side.

In contrast, the variance of max opening velocity showed a trend toward being smaller on the deviated side (*p* = 0.035), but this difference did not remain significant after FDR correction (q = 0.070). No significant difference was observed in the variance of cycle duration.

The effect sizes for velocity-related parameters were generally small to moderate, indicating limited to moderate clinical relevance of the observed differences.

### 3.3. Prevalence of MRI-Based Structural Features of the Temporomandibular Joint

Table 4 shows a comparison of the prevalence of MRI-based TMJ structural features between the deviated and non-deviated sides.

The prevalence of MRI-based structural features of the temporomandibular joint was higher on the deviated side than on the non-deviated side, and McNemar’s exact test demonstrated a significant difference (66.7% vs. 33.3%, *p* = 0.0156, q = 0.042).

Among the discordant cases, seven patients showed these imaging features only on the deviated side, whereas none showed these features only on the non-deviated side. This asymmetry was statistically significant and showed a moderate to large effect size (φ = 0.58).

## 4. Discussion

### 4.1. Oral Function and Path and Velocity of Masticatory Movement

Chewing on the deviated side showed a smaller masticatory path width and a smaller closing angle than chewing on the non-deviated side. These findings indicate that chewing on the deviated side was characterized by a chopping-type masticatory movement pattern with a more vertical closing path, whereas chewing on the non-deviated side exhibited a grinding-type pattern with a more lateral closing path. These tendencies are generally consistent with previous reports [35,36]. In patients with facial asymmetry, the molar occlusion on the deviated side often presents with crossbite due to morphological reasons. Previous studies have reported that cases with first molar crossbite frequently exhibit a reverse-type masticatory movement pattern; therefore, a similar mechanism may explain the present findings [37,38].

In general, regarding the relationship between the masticatory path and masticatory performance, grinding-type masticatory movement characterized by a lateral closing path has been reported to be more efficient than chopping-type movement characterized by a vertical closing path [39]. From a biomechanical perspective, the combination of a more vertical masticatory pattern with greater occlusal contact area, occlusal force, and movement stability on the deviated side may reflect a functional adaptation rather than representing a contradiction.

In patients with facial asymmetry, the more vertical (chopping-type) masticatory movement observed on the deviated side may reduce lateral mandibular displacement and, under an occlusal relationship characterized by a reversed functional cusp relationship, facilitate more direct force transmission during occlusion, thereby contributing to improved mechanical efficiency and movement stability. In contrast, the grinding-type masticatory movement observed on the non-deviated side, which involves greater lateral displacement, may increase variability in the direction of force application and reduce the consistency of occlusal contacts [37]. However, because the present study did not assess the detailed occlusal pattern of each individual case, this interpretation remains hypothetical and should be made with caution.

Therefore, the observed vertical pattern on the deviated side may represent a movement strategy that prioritizes stability and efficient force transmission under the existing occlusal and morphological conditions.

In addition to functional and biomechanical factors, the observed vertical masticatory pattern on the deviated side may also be influenced by underlying skeletal morphology. In patients with facial asymmetry, the deviated side is often associated with structural differences such as a shorter mandibular ramus, altered posterior facial height, or asymmetry of the mandibular body and symphysis. These morphological characteristics may restrict lateral mandibular movement and favor a more vertical closing path during mastication.

Furthermore, asymmetrical skeletal relationships may influence occlusal plane inclination and occlusal contact patterns, which in turn can affect the direction and stability of masticatory movement. Therefore, the vertical masticatory pattern observed on the deviated side may reflect not only functional adaptation but also mechanical constraints imposed by underlying skeletal morphology.

However, the deviated side showed greater occlusal contact area and occlusal force, as well as greater stability of chewing speed and masticatory path. The finding that occlusal contact area and occlusal force were greater on the deviated side is consistent with previous reports [1,2].

Favorable occlusal relationships have been associated with reduced variability in masticatory movement [14], and increases in occlusal contact area and occlusal force have also been associated with improved stability of masticatory movement. Furthermore, the acquisition of functionally stable occlusion and occlusal support has been reported to shorten masticatory muscle activity time during chewing and suppress excessive muscle activity during masticatory movement [15,16].

In addition, sensory input from periodontal mechanoreceptors during occlusion has been reported to affect masticatory rhythm [12]. Therefore, on the deviated side, where occlusal contact area is larger and occlusal support is relatively more stable, more stable masticatory muscle activity may contribute to greater stability in chewing speed.

Another possible explanation for the greater stability of masticatory movement on the deviated side is that this side may be associated with preferential use during chewing rather than representing a definitive habitual chewing side in these patients. Although mastication is generally a bilateral function, unilateral chewing preference commonly develops in daily life, even in healthy individuals [19,40]. Moreover, previous studies have reported that masticatory movement on the habitual chewing side is performed with greater reproducibility and with less variability in masticatory rhythm and path [11,18,41].

Greater occlusal contact area and occlusal force on the deviated side suggest richer sensory input from periodontal mechanoreceptors during chewing. Since input from periodontal mechanoreceptors has been reported to be involved in the regulation of masticatory muscle activity [13], the deviated side, with greater occlusal contact area and occlusal force, may provide a more favorable functional environment for mastication and may be associated with preferential use during chewing rather than indicating a definitive habitual chewing side.

In addition, the development of a habitual chewing side has also been reported to be associated with occlusal contact conditions, occlusal force, and masticatory performance, and previous reports are consistent with this interpretation [40,42].

Although the habitual chewing side was not directly evaluated in the present study, it is possible that the deviated side may be associated with a tendency toward preferential chewing behavior; however, this interpretation should be considered with caution, as habitual chewing preference was not directly assessed in the present study.

These considerations should be interpreted in light of the study design, particularly its retrospective and cross-sectional nature. In addition, habitual chewing behavior was not directly assessed.

From a clinical perspective, the observed moderate effect sizes suggest that the differences between the deviated and non-deviated sides may not only be statistically significant but also potentially functionally relevant. In particular, the greater occlusal contact area and stability of masticatory movement on the deviated side may reflect a tendency toward functional asymmetry.

### 4.2. Prevalence of MRI-Based Structural Features of the Temporomandibular Joint

The prevalence of MRI-based structural features of the temporomandibular joint was significantly higher on the deviated side. These findings suggest that such imaging features may be preferentially located on the deviated side. Among discordant cases, these features were more frequently observed only on the deviated side, which is consistent with previous studies reporting a higher prevalence of disc displacement and condylar deformity on the deviated side in patients with facial asymmetry [6,8,9,43].

The present study also showed greater occlusal contact area and occlusal force on the deviated side, suggesting that functional differences between the two sides may be associated with the presence of these imaging-based structural features [44,45,46].

However, temporomandibular disorders are multifactorial in nature and cannot be explained solely by mechanical factors [12,47,48]. Therefore, although the present findings suggest that these imaging-based structural features may be more frequently observed in the temporomandibular joint on the deviated side, further investigation is needed to clarify the underlying mechanisms.

A more detailed subclassification of temporomandibular joint structural features—such as disc displacement with reduction, disc displacement without reduction, and osteoarthritic changes—may provide additional insights into side-specific patterns. Such a granular approach could help clarify whether different types of joint pathology are differentially associated with functional asymmetry.

In addition, the absence of a control group (e.g., healthy subjects without facial asymmetry) limits the ability to determine whether these findings are specific to patients with facial asymmetry.

It should be noted that the present findings are based on imaging assessments and do not represent a comprehensive clinical diagnosis of temporomandibular disorders, which requires both clinical and imaging evaluation according to established diagnostic criteria, such as the Diagnostic Criteria for Temporomandibular Disorders (DC/TMD) [49,50].

The imaging-based structural features evaluated in this study do not correspond to a clinical diagnosis of temporomandibular disorders as defined by the Diagnostic Criteria for Temporomandibular Disorders (DC/TMD) [49,50], which requires both clinical examination and patient-reported symptoms.

Therefore, the relationship between these imaging findings and clinical symptoms remains unclear, and the higher prevalence observed on the deviated side should be interpreted with caution.

### 4.3. Limitations

One limitation of this study is that the pattern and stability of the masticatory path may be influenced by the individual occlusal scheme of each patient [51]. Additionally, occlusal force may have been affected by side-to-side differences in masticatory muscle activity between the deviated and non-deviated sides.

Moreover, although no significant difference in masticatory performance was observed between the two sides, the values obtained using the color-changeable chewing gum in this study were higher on both sides than those reported in previous studies [52,53]. This suggests that the task load used to evaluate masticatory performance may have been relatively low, making it difficult to detect side-to-side differences sufficiently. This suggests that the task load used to evaluate masticatory performance may have been relatively low, making it difficult to detect side-to-side differences sufficiently. This limitation may also contribute to a ceiling effect, particularly in subjects with relatively preserved masticatory function, thereby reducing the sensitivity of the method to detect subtle functional differences.

A further limitation relates to the evaluation of masticatory movement stability. The stability of masticatory movement was evaluated using the variance calculated from 10 chewing cycles. Although this approach is commonly used in masticatory movement analysis, a limited number of cycles may not fully capture intra-individual variability, and a larger number of cycles may provide a more robust assessment.

In addition, this study was designed as a retrospective cross-sectional study based on clinical records obtained at a single institution. Therefore, selection bias cannot be excluded, and the study population may not be representative of the general population of patients with facial asymmetry.

Although standardized clinical protocols were used for data collection, variability in measurement conditions and patient performance during functional tests may have influenced the results. In particular, potential confounding factors, such as the severity of facial asymmetry, skeletal classification, and occlusal characteristics, were not fully controlled in this study.

Moreover, side-specific skeletal parameters, such as the magnitude and direction of menton deviation or differences between the short and long sides, were not quantitatively analyzed in relation to masticatory movement variables in the present study. Therefore, the potential relationship between the severity of skeletal asymmetry and functional characteristics of masticatory movement remains unclear.

Future studies should investigate these associations to provide a more comprehensive understanding of the interplay between skeletal morphology and masticatory function. These unmeasured factors may have influenced the observed side-to-side differences. In the context, causal relationships between the greater stability of masticatory rhythm and masticatory path on the deviated side and oral functional parameters cannot be definitively established.

Future studies should include post-treatment data and conduct longitudinal analyses to clarify these relationships more clearly.

Furthermore, the classification of the deviated and non-deviated sides was based solely on menton deviation on posteroanterior cephalograms and did not account for other factors related to functional or occlusal asymmetry, such as dental midline discrepancies, occlusal cant, or unilateral crossbite. Therefore, the present classification may not fully capture the complexity of functional asymmetry, which is likely influenced by multiple morphological and occlusal factors.

Similarly, the definition of facial asymmetry in this study was based primarily on mandibular deviation as indicated by menton displacement and did not distinguish between maxillary and combined dentofacial asymmetry.

Furthermore, facial asymmetry represents a heterogeneous condition that may include mandibular-, maxillary-, or occlusal (e.g., crossbite-related) asymmetry. These different phenotypes may be associated with distinct functional characteristics; however, such distinctions were not evaluated in the present study. Therefore, the present findings may not fully reflect the complexity of skeletal and dental asymmetry.

An additional limitation is that inter- and intra-observer reliability for the interpretation of CT and MRI findings was not formally assessed in this study. Therefore, the potential influence of observer variability cannot be excluded, and future studies should include reliability analysis to validate imaging-based evaluations.

Additionally, temporomandibular joint structural features were analyzed as a combined category in the present study. More detailed subclassification, such as distinguishing between disc displacement with reduction, disc displacement without reduction, and osteoarthritic changes, was not performed due to the limited sample size. Therefore, potential differences in side-specific patterns among these subtypes could not be evaluated.

Another consideration is that the relatively small sample size of this study may limit the statistical power, particularly for detecting small or highly variable effects in functional parameters such as masticatory movement variability. Moreover, the effect size assumed in the a priori power analysis may have been optimistic given the heterogeneity of these measurements. Therefore, the generalizability of the findings may be restricted.

Furthermore, given the relatively large number of statistical comparisons performed, the possibility of type I error cannot be excluded. Accordingly, the findings should be interpreted with caution. The present study should be considered exploratory, and further studies with larger sample sizes are required to confirm these results.

Finally, the habitual chewing side was not directly evaluated in this study. Therefore, the relationship between the deviated side and preferential chewing behavior remains speculative and requires further investigation. This limitation is particularly relevant when interpreting the greater stability of masticatory movement on the deviated side, as alternative explanations cannot be excluded.

## 5. Conclusions

In this cross-sectional study, patients with dentofacial deformity and facial asymmetry exhibited side-to-side differences in masticatory movement and oral function between the deviated and non-deviated sides. The deviated side demonstrated greater occlusal contact area, occlusal force, and stability of masticatory movement. These findings suggest that the deviated side may be associated with side-dependent differences in masticatory function, although the underlying mechanisms, including potential behavioral factors such as chewing preference, remain unclear. Understanding these side-to-side functional differences may be useful for the clinical evaluation of oral function and temporomandibular joint structural features assessed by imaging and may provide clinically relevant information for treatment planning in patients with dentofacial deformity and facial asymmetry.

## Figures and Tables

**Table 1:** Comparison of oral function between the deviated and non-deviated sides.

Oral Function	Deviated Side (Mean ± SD)	Non-Deviated Side (Mean ± SD)	Cohen’s d [95% CI]	*p*-Value	FDR-Adjusted *p* (q)
Occlusal contact area (mm^2^)	7.44 ± 3.24	5.20 ± 2.96	0.91 [0.39, 1.41]	0.001 ^a^	0.016 *
Occlusal force (N)	313.81 ± 136.59	242.85 ± 133.88	0.67 [0.19, 1.13]	0.006 ^b^	0.019 *
Masticatory performance (ΔE)	42.52 ± 5.80	42.93 ± 5.95	−0.14 [−0.57, 0.29]	0.211 ^a^	0.338

* Data are presented as mean ± standard deviation. Effect sizes (Cohen’s d) and 95% confidence intervals (CIs) were calculated from paired *t*-test statistics. *p*-values: ^a^ Wilcoxon signed-rank test; ^b^ paired *t*-test. Adjusted *p*-values (q-values) were calculated using the Benjamini–Hochberg false discovery rate (FDR) method across all 16 statistical tests. * indicates statistical significance at q < 0.05. All units are consistently presented for each parameter (e.g., mm, mm^2^, and degrees), as appropriate.

**Table 2:** Comparison of masticatory path and path stability between the deviated and non-deviated sides.

Parameter	Deviated Side (Mean ± SD)	Non-Deviated Side (Mean ± SD)	Cohen’s d [95% CI]	*p*-Value	FDR-Adjusted *p* (q)
Closing angle (°)	12.55 ± 15.94	22.90 ± 13.56	−0.68 [−1.15, −0.20]	0.005 ^b^	0.019 *
Max lateral amplitude (mm)	3.75 ± 1.39	4.40 ± 1.71	−0.56 [−1.01, −0.09]	0.019 ^b^	0.043 *
Cycle axis angle (°)	10.00 ± 8.66	9.90 ± 6.49	0.01 [−0.42, 0.44]	0.964 ^b^	0.964
Variance of closing angle	7.91 ± 11.97	9.04 ± 9.40	−0.07 [−0.50, 0.36]	0.322 ^a^	0.468
Variance of max lateral amplitude	1.16 ± 0.80	1.58 ± 1.31	−0.37 [−0.81, 0.08]	0.375 ^a^	0.500
Variance of cycle axis angle	45.37 ± 41.56	77.66 ± 49.38	−0.64 [−1.10, −0.16]	0.002 ^a^	0.016 *

Path stability was evaluated using the variance across chewing cycles. * Data are presented as mean ± standard deviation. Effect sizes (Cohen’s d) and 95% confidence intervals (CIs) were calculated from paired *t*-test statistics. *p*-values: ^a^ Wilcoxon signed-rank test; ^b^ paired *t*-test. Adjusted *p*-values (q-values) were calculated using the Benjamini–Hochberg false discovery rate (FDR) method across all 16 statistical tests. * indicates statistical significance at q < 0.05. All units are consistently presented for each parameter (e.g., mm, and degrees), as appropriate.

**Table 3:** Comparison of masticatory velocity and velocity stability between the deviated and non-deviated sides.

Parameter	Deviated Side (Mean ± SD)	Non-Deviated Side (Mean ± SD)	Cohen’s d [95% CI]	*p*-Value	FDR-Adjusted *p* (q)
Max closing velocity	81.19 ± 31.47	81.64 ± 29.40	−0.03 [−0.46, 0.40]	0.890 ^b^	0.964
Max opening velocity	91.26 ± 30.34	88.89 ± 32.07	0.26 [−0.17, 0.70]	0.181 ^a^	0.322
Cycle duration	0.84 ± 0.17	0.84 ± 0.13	−0.02 [−0.45, 0.41]	0.931 ^b^	0.964
Variance of max closing velocity	216.71 ± 112.15	343.32 ± 231.83	−0.64 [−1.10, −0.16]	0.006 ^a^	0.019 *
Variance of max opening velocity	386.85 ± 353.34	524.38 ± 488.12	−0.57 [−1.03, −0.11]	0.035 ^a^	0.070
Variance of cycle duration	0.011 ± 0.010	0.046 ± 0.122	−0.28 [−0.71, 0.16]	0.870 ^a^	0.964

Velocity stability was evaluated using variance.* Data are presented as mean ± standard deviation. Effect sizes (Cohen’s d) and 95% confidence intervals (CIs) were calculated from paired *t*-test statistics. *p*-values: ^a^ Wilcoxon signed-rank test; ^b^ paired *t*-test. Adjusted *p*-values (q-values) were calculated using the Benjamini–Hochberg false discovery rate (FDR) method across all 16 statistical tests. * indicates statistical significance at q < 0.05.

**Table 4:** Comparison of MRI-based TMJ structural feature prevalence between the deviated and non-deviated sides.

	Non-Deviated (+)	Non-Deviated (−)	Total
Deviated (+)	7	7	14
Deviated (−)	0	7	7
Total	7	14	21

Data are presented as the number of subjects. Side differences were evaluated using the exact McNemar test (two-sided). Discordant pairs: deviated-only positive = 7, non-deviated-only positive = 0. Raw *p* = 0.0156; FDR-adjusted *p* (q) = 0.042. The effect size (phi coefficient) was 0.58, indicating a moderate to large association.

## Data Availability

The original contributions presented in this study are included in the article/Supplementary Material. Further inquiries can be directed to the corresponding author.

## Supplementary Materials

The following supporting information can be downloaded at https://www.mdpi.com/article/10.3390/diagnostics16091274/s1, File S1: All the data analyzed in this study.
